# A Global Survey of the Full-Length Transcriptome of *Apis mellifera* by Single-Molecule Long-Read Sequencing

**DOI:** 10.3390/ijms24065827

**Published:** 2023-03-18

**Authors:** Shuang-Yan Zheng, Lu-Xia Pan, Fu-Ping Cheng, Meng-Jie Jin, Zi-Long Wang

**Affiliations:** 1 College of Animal Science and Technology, Jiangxi Agricultural University, Nanchang 330045, China; 2 Sino-German Joint Research Institute, Nanchang University, Nanchang 330047, China; 3 Honeybee Research Institute, Jiangxi Agricultural University, Nanchang 330045, China; 4 Jiangxi Province Key Laboratory of Honeybee Biology and Beekeeping, Jiangxi Agricultural University, Nanchang 330045, China

**Keywords:** PacBio sequencing, alternative splicing, single-molecule sequencing, alternative polyadenylation site, differentially expressed transcript

## Abstract

As important pollinators, honey bees play a crucial role in both maintaining the ecological balance and providing products for humans. Although several versions of the western honey bee genome have already been published, its transcriptome information still needs to be refined. In this study, PacBio single-molecule sequencing technology was used to sequence the full-length transcriptome of mixed samples from many developmental time points and tissues of *A. mellifera* queens, workers and drones. A total of 116,535 transcripts corresponding to 30,045 genes were obtained. Of these, 92,477 transcripts were annotated. Compared to the annotated genes and transcripts on the reference genome, 18,915 gene loci and 96,176 transcripts were newly identified. From these transcripts, 136,554 alternative splicing (AS) events, 23,376 alternative polyadenylation (APA) sites and 21,813 lncRNAs were detected. In addition, based on the full-length transcripts, we identified many differentially expressed transcripts (DETs) between queen, worker and drone. Our results provide a complete set of reference transcripts for *A. mellifera* that dramatically expand our understanding of the complexity and diversity of the honey bee transcriptome.

## 1. Introduction

Honey bees are important pollinating insects, playing a crucial role in promoting biodiversity and maintaining ecological balance. They are characterized by high reproductive performance, short generation cycles, easiness of artificial breeding and division of labor. *A. mellifera* is the world’s most widely bred honey bee species with the excellent ability to collect large nectar sources and gums [1]. The first version of the *A. mellifera* complete genome sequence was published in 2006 [2]; since then, several versions of genome sequences have been released on Genbank. So far, the genomic sequences [2,3], transcriptome [4,5] and genetic linkage map [6,7,8] of *A. mellifera* have been studied extensively.

Transcriptomes provide valuable gene transcription information, including alternative splicing (AS) events, long non-coding RNAs (lncRNAs) and alternative polyadenylation (APA) sites. This is especially useful for species lacking genome sequence information. Alternative splicing of pre-mRNAs is an effective means of regulating gene expression and increasing proteome diversity in higher eukaryotes [9,10]. Alternative splicing is coordinately controlled by multiple transcriptional and post-transcriptional regulatory mechanisms, and is involved in many biological processes including growth, development, signal transduction and response to stress [11]. The abundance of alternative splicing events reaches >95–100% in human genes [12], 63% in mouse genes [13] and 20% to 60% in plant intron genes [14,15,16,17]. Long non-coding RNAs are abundant in organisms and are important regulators of gene expression [18]. Alternative polyadenylation events are a ubiquitous form of gene expression regulation in eukaryotic cells and they play an important role in many biological processes, including development, proliferation, differentiation and neuronal activation [19].

High-throughput transcriptome sequencing technology has been widely used to obtain transcript sequences and to analyze gene expression differences [20,21]. However, the second-generation sequencing technology requires the assembly of short reads (100–150 bp) that lead to low accuracy in the subsequent gene structure analysis, such as alternative splicing and gene fusion, because of the incomplete and inaccurate assembly process. In addition, the sequences of complex regions, such as highly repetitive and high GC content sequences, cannot be assembled. A third-generation sequencing platform can obtain high-quality full-length transcripts directly without PCR amplification and assembly during the sequencing and data analysis. Based on these full-length transcripts, we can precisely identify AS events, APA sites and fusion genes from the transcriptome of an organism.

At present, there are two representative third-generation sequencing (TGS) technologies: the single-molecule real-time (SMRT) sequencing technology from Pacific Biosciences (PacBio) [22] and Oxford Nanopore Technology (ONT) [23]. The SMRT sequencing method uses zero-mode waveguide (ZMW) technology that enables direct observation of the synthesis of DNA strands by a single DNA polymerase molecule. On the other hand, Oxford nanopore sequencing can directly identify a single nucleotide when a single DNA or RNA molecule passes through the nanopore (nanometer size) by electrophoresis. Both technologies are similar in terms of reading length, error rate and throughput and both can produce long reads that exceed most transcripts. They are thus widely used for genome assembly [24,25] and the identification of full-length transcripts [26,27].

The full landscape of the western honey bee transcriptome is not well understood. In this study, we attempt to build a catalog of all the full-length transcripts of *A. mellifera* and explore the complexity of the transcriptome using TGS technologies. To this end, we sequenced the full-length transcriptome of three mixed samples of queen and drone and worker of *A. mellifera* using the SMRT sequencing technique. The results of this study will improve honey bee genome sequence analysis and provide a complete set of reference transcriptomes for gene function study in *A. mellifera*.

## 2. Results

### 2.1. Sequencing Results and Data Assembly

Sequencing using the PacBio sequencing platform resulted in 1,100,885, 1,042,133 and 1,126,347 polymerase reads for the queen, worker and drone samples, respectively (Table 1, Figure S1). From these polymerase reads, 43,491,193, 40,055,924 and 40,663,357 subreads as well as 795,183, 735,061 and 792,515 circular consensus sequences (CCSs) were obtained. The final numbers of full-length non-chimeric (FLNC) reads were 556,221, 514,591 and 546,907, with an average length of 1504, 1511 and 1651 bp (Figure S2). The full-length transcripts accounted for 83.10%, 80.04% and 83.58% of the total clean reads in the three samples, respectively. After eliminating redundant FLNC sequences from the same transcript, 116,535 non-redundant FLNC sequences from 30,045 genes were obtained for subsequent analysis (Table S1).

### 2.2. Functional Annotation of Transcripts

**Figure 1:**
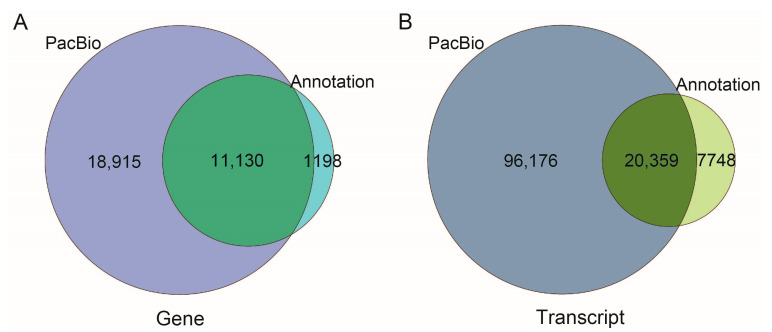
Comparison of transcripts (**A**) and genes (**B**) generated by the PacBio sequencing and *A. mellifera* genome annotation.

The 116,535 transcripts were compared with the 12,328 known annotated genes in the *A. mellifera* reference genome (Genbank accession No.: GCA_003254395.2). A total of 11,130 gene loci and 20,359 transcripts overlapped with the genome annotation; 18,915 gene loci and 96,176 transcripts were newly identified in this study. These new transcripts accounted for 82.53% of the total transcripts (Figure 1A,B). Of the new transcripts, 76,018 were new transcripts from known genes and 20,158 were from new genes. After blast comparison of the 116,535 transcripts with the NR, GO, KO, KOG and Swiss-Prot databases, 92,477 transcripts were annotated (Tables S2 and S3). For the 18,915 new genes, 2967 genes were annotated.

### 2.3. Unique Transcripts from Queen, Worker and Drone Data

**Figure 2:**
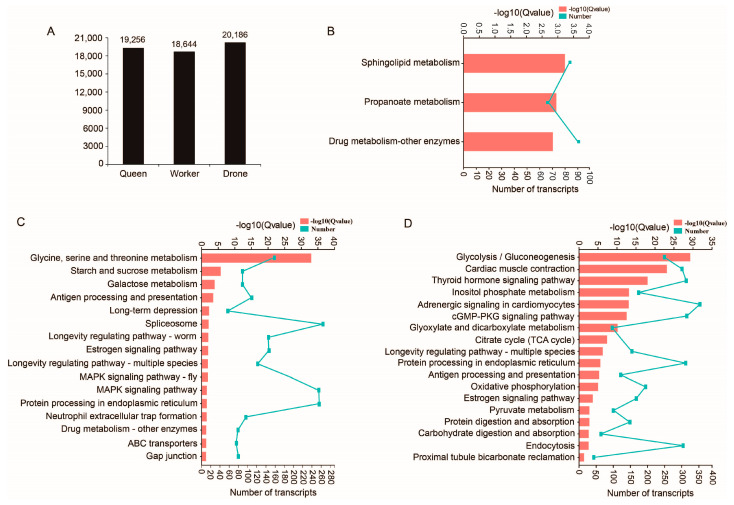
Unique transcripts isolated from queen, worker and drone datasets. (**A**) Numbers of unique transcripts in queen, worker and drone datasets. (**B**–**D**) show the significantly enriched KEGG pathways for queen-, worker- and drone-unique transcripts, respectively.

There were 19,256, 18,644 and 20,186 transcripts unique to the queen, worker and drone datasets with an average length of 1828 bp, 1875 bp and 1830 bp, respectively (Figure 2A, Table S4). GO analysis of these unique transcripts showed that queen-unique transcripts were primarily enriched in 249 GO terms (*p* < 0.05, Table S5), worker-unique transcripts in 376 GO terms (*p* < 0.05), and drone-unique transcripts in 337 GO terms (*p* < 0.05). The largest GO terms in queen, worker and drone were “catalytic activity”, “binding” and “ion binding”, respectively. KEGG analysis showed that queen-unique transcripts were significantly enriched in three pathways (q < 0.05, Figure 2B), drone-unique transcripts in 18 pathways (q < 0.05, Figure 2C), and worker-unique transcripts in 16 pathways (q < 0.05, Figure 2D). Of these, many pathways were related to carbohydrate metabolism. Four pathways related to honey bee caste differentiation were significantly enriched in the worker, including the “Longevity regulating pathway–worm”, “Longevity regulating pathway–multiple species”, “MAPK signaling pathway–fly” and the “MAPK signaling pathway”.

### 2.4. Alternative Splicing

**Figure 3:**
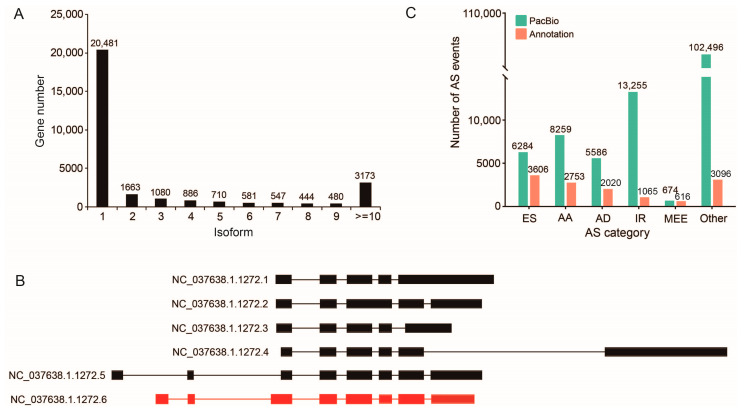
The alternative splicing events identified from PacBio honey bee transcriptome. (**A**) Distribution of splice isoforms of genes. (**B**) The exon/intron structure of the six isoforms of the *Mob3* gene. The filled boxes represent exons and lines represtent introns. The isoform in red was obtained by genome annotation. (**C**) The comparison of each alternative splicing type between this PacBio data and the NCBI reference transcript set.

Gene transcript analysis revealed that 20,481 (68.17%) genes contained one isoform, 1663 (5.54%) contained two isoforms, and 3173 (10.56%) contained >10 isoforms (Figure 3A). The *A. mellifera Mob3* gene was found to contain six isoforms, though it was only annotated as one in the reference genome (Figure 3B).

The alternative splicing events in the three mixed samples were analyzed, and 25,289, 62,103 and 42,352 alternative splicing events were detected in the queen, worker and drone datasets, respectively (Figure S3). By combining the data of the three RNA samples, a total of 136,554 AS events originating in 6435 genes were detected. Of the five major AS types, intron retention (IR) was the predominant type, originating in 4074 genes (Figure 3C). In contrast, only 13,156 AS events were detected in the NCBI reference transcript set of *A. mellifera* (Figure 3C); this is less than the AS events obtained in this study by an order of magnitude.

**Figure 4:**
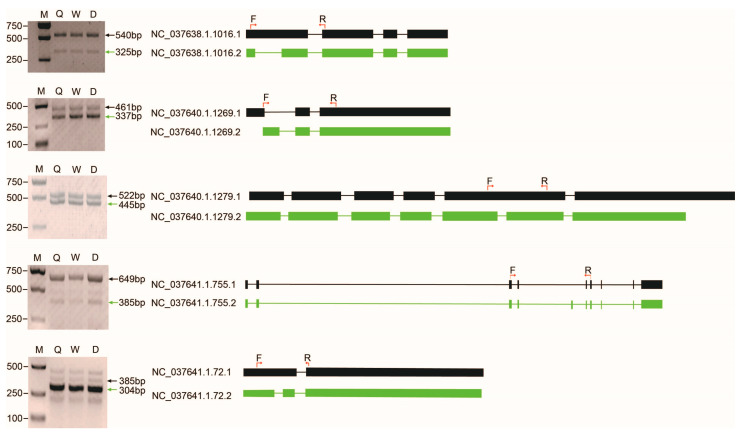
Verification of the alternative splicing events in five genes by RT-PCR and Sanger sequencing. The exon/intron structure of each isoform of each gene is shown in the right panel. The filled boxes represent exons and the lines represent introns. Different isoforms of each gene are displayed in green or black. The locations of the PCR primers are shown with red arrows on the first isoform of each gene. M: marker, Q: queen, W: worker, D: drone.

Five genes were randomly selected to validate the accuracy of AS events using RT-PCR. The results indicated that the size of each amplified fragment was consistent with what was expected (Figure 4).

### 2.5. APA Sites

**Figure 5:**
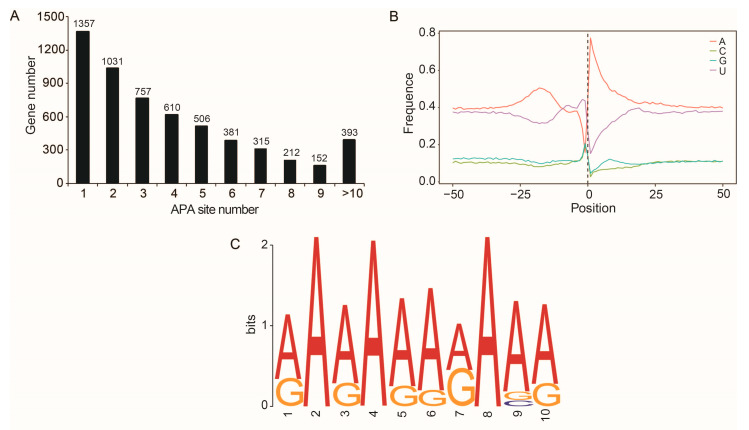
Alternative polyadenylation sites identified from the PacBio honey bee transcriptome. (**A**) Distribution of the number of APA sites per gene. (**B**) Nucleotide composition 50 bp upstream and downstream of the poly (**A**) sites. (**C**) A conserved element near the poly (**A**) sites predicted by MEME analysis.

In the queen, worker and drone datasets, 14,491, 14,021 and 15,213 APA loci from 5364, 5262 and 5650 gene loci were identified, respectively (Figure S4). Based on the 116,535 PacBio transcripts, a total of 23,376 APA events at 5714 gene loci were identified, of which 5321 (93.12%) genes had 1–9 poly (A) sites and 393 (6.88%) genes had more than 10 poly (A) sites, with an average of 4.09 poly (A) sites per gene (Figure 5A). The nucleic acid composition 50 bp upstream and downstream of the poly (A) sites were enriched with uracil (U) and adenine (A) (Figure 5B), and a conserved element (AAAAAAAAAA) was over-presented in this region (Figure 5C).

### 2.6. Long Non-Coding RNAs

**Figure 6:**
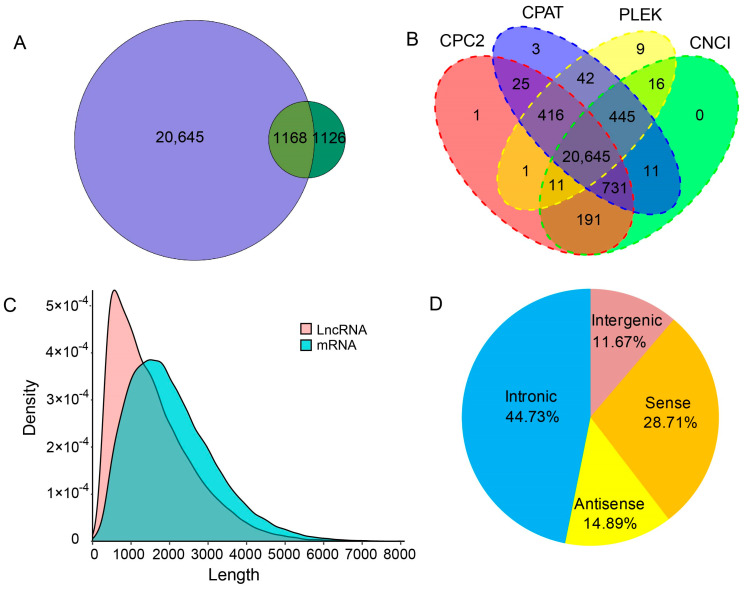
LncRNAs identified from the PacBio honey bee transcriptome. (**A**) Comparison of the lncRNAs identified in this study with those from genome annotation. (**B**) The Venn diagram of the number of lncRNAs predicted by CPC2, CPAT, PLEK and CNCI. (**C**) The length density distribution of lncRNAs and mRNAs. (**D**) Proportions of the four kinds of lncRNAs.

A total of 21,813 lncRNAs were identified from the 116,535 transcripts and, of these, 1168 lncRNAs overlapped with known lncRNAs from genome annotation (Figure 6A). A total of 20,645 lncRNAs were novel lncRNAs predicted by CPC/CNCI/CPAT/Pfam analysis (Figure 5B). The average length of all the lncRNAs was significantly shorter than that of the mRNAs (Figure 6C). All the lncRNAs were classified into four types according to their positional relationship in the *A. mellifera* genome. Finally, 9758 (44.73%), 6262 (28.71%), 3248 (14.89%) and 2545 (11.67%) lncRNAs were classified as located in the intronic region, sense, antisense or intergenic region, respectively, where intronic region lncRNA was the dominant type (Figure 6D).

### 2.7. DETs and DEGs between the Three Honey Bee Castes

Based on full-length transcripts obtained from this study, we used the RNA-seq data from 2-day and 4-day larvae of the three honey bee castes published previously [28] to explore whether expression differences between the castes are more evident at the transcript level.

**Figure 7:**
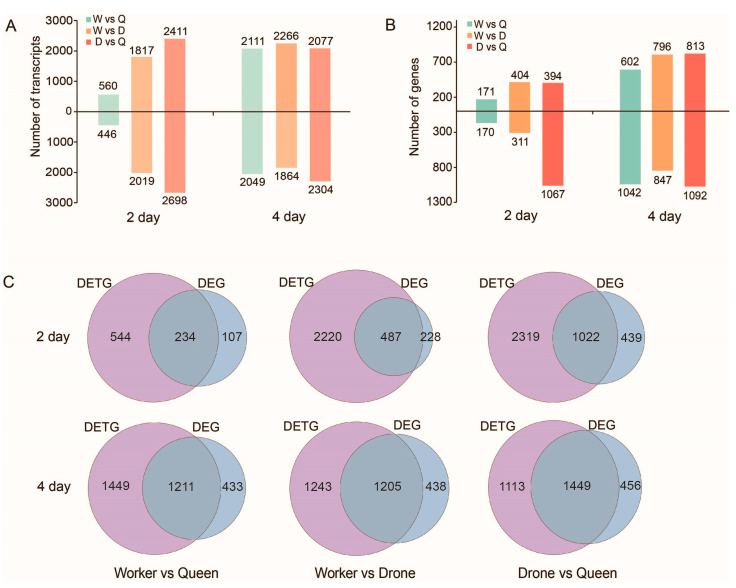
The DETs and DEGs between castes at larvae stage. The DETs (**A**) and DEGs (**B**) among queen, worker and drone at 2-day and 4-day larvae stages. (**C**) The overlapped genes between DETGs and DEGs.

When comparing queen larvae vs. worker larvae, worker larvae vs. drone larvae and queen larvae vs. drone larvae, at the 2-day larval stage, there were 1006, 3836 and 5109 DETs related to 778, 2707 and 3341 genes as well as 341, 715 and 1461 DEGs (Figure 7A,B; Table S6). At the 4-day larval stage, there were 4160, 4130 and 4381 DETs related to 2660, 2448 and 2562 genes as well as 1644, 1643 and 1905 DEGs (Figure 7A,B; Table S6). Many of the DETs were related to genes involved in caste differentiation or sex determination of honey bees, such as *Igf1*, *Fem* and *Amdsx* (Table S6). The numbers of overlapping genes between the DETGs and DEGs occupied 17.99–56.56% and 68.11–76.06% of the DETGs and DEGs, respectively (Figure 7C).

## 3. Discussion

Although the genome sequence of the western honey bee *A. mellifera*, a very important Hymenoptera model organism, has been published [2], its transcriptome features and mRNA structure have not been analyzed in depth. With the improvement of sequencing technologies, SMRT provides the possibility to profile the *A. mellifera* transcriptome. Compared with second-generation sequencing technologies, the TGS technologies, represented by PacBio’s SMRT sequencing, can directly sequence the cDNA to obtain full-length transcripts. In this study, 116,535 transcripts were obtained by PacBio sequencing of the honey bee transcriptome; this is 4.15 times the number of transcripts recorded in NCBI annotations. Also, many novel or previously unidentified protein-coding genes were identified. These transcript datasets represent the largest and most complete set of full-length transcripts of the western honey bee, containing a broad diversity of transcript isoforms that are well-suited for studying alternative splicing, gene fusion, etc.

Alternative splicing is an important transcriptional process in eukaryotes and one of the methods that produce different transcriptional isoforms that can significantly expand the diversity of gene function [9,10]. In this study, we detected 136,554 AS events from 6435 genes, occupying 21.42% of genes identified in this study; this is similar to the ratio reported in *Drosophila* [29] and higher than that in *Bombyx mori* [30] and *Plutella xylostella* [31]. These results suggest that AS is a widely used mRNA post-transcriptional process mechanism in honey bees. 

Selection of different polyadenylation sites in pre-mRNAs will result in isoforms with different lengths of coding or 3′ untranslated regions, further increasing transcriptome diversity and thus finely regulating mRNA stability, localization and translation efficiency. About half of the genes in *Drosophila* [32], worms [33] and zebrafish [34] have more than two APA sites. Recent studies using high-throughput sequencing have shown that APA regulates gene expression through multiple mechanisms in plants and animals [35,36,37]. In the present study, we identified a large number of APA loci in honey bees; moreover, genes with more than two APA loci account for 76.25% of the total number of APA loci-related genes in the honey bee transcriptome. Our findings suggest that APA is one of the important processes for promoting transcriptome diversity of honey bees.

In this study, we identified a large number of lncRNAs, of which 94.65% were newly identified lncRNAs. In mammals, lncRNAs can account for up to 4–9% of total RNA, whereas protein-coding RNAs account for only 1% of total RNA [38]. This suggests that the prevalence of lncRNAs from both coding and noncoding genes is higher in honey bees than previously thought. Interestingly, lncRNAs from intron regions were the largest type, while in other insects lncRNAs from intergenic regions are the most abundant type [39,40,41,42]. This suggests that introns in honey bee genes may play an important role in gene transcription regulation through lncRNAs. Recent studies have shown that lncRNAs are numerous and are involved in many important biological processes, including X chromosome silencing [43,44], chromosome structure and genome rearrangements [45,46,47], imprinting [48,49], transcriptional activation and suppression [50,51]. The large number of lncRNAs identified in this study may also be extensively involved in various biological processes in honey bees.

In addition, based on the transcripts obtained in this study, we identified a large number of DETs among the three honey bee castes using RNA-seq data. The number of DET-related genes is 2.7 times the number of DEGs reported in a previous study [28]. Moreover, the overlapping genes between the DETGs and DEGs just occupy a middle proportion of the DETGs. It suggests that many genes showed differential expression between the honey bee castes at some splicing isoforms and not at the whole gene level. These findings suggest that phenotypic differences between the three honey bee castes are more likely to be regulated at the transcript isoform level, rather than at the gene level. 

In conclusion, this study greatly expanded the data set of *A. mellifera* gene transcripts, providing a basis for discovering new genes and transcripts and for conducting gene function studies in *A. mellifera*. 

## 4. Materials and Methods

### 4.1. Sample Collection

The experimental colonies of *A. mellifera* were raised by the Honeybee Research Institute, Jiangxi Agricultural University, Nanchang, Jiangxi province, China (28.46 °N, 115.49 °E). For the worker samples, a fertilized queen from a healthy colony was controlled on an empty comb to lay eggs for 6 h. Then, ten developmental time points and eight organs/tissues of newly emerged workers were sampled. The same queen and colony were used for collecting queen samples. Similarly, the queen was allowed to lay eggs on an empty worker comb for 6 h; after 72 h, 1-day-old larvae in the worker cells were moved to queen cells to cultivate queens. Ten developmental time points and eight organs/tissues of newly emerged queens were sampled. For the drone samples, an unfertilized queen was allowed to lay eggs on an empty drone cell comb for 6 h. Then, ten developmental time points and eight organs/tissues of newly emerged drones were sampled. The developmental time points and organs/tissues sampled are listed in Table S7. All the samples were frozen and stored in liquid nitrogen for further use.

### 4.2. Library Construction and Sequencing

The total RNA of each sample was extracted separately using the TRIzol^TM^ Regent (Invitrogen, Carlsbad, CA, USA). The quality of the RNA samples was measured using a Nanodrop 2000 (Thermo Fisher Scientific, Wilmington, DE, USA) and the Agilent Bioanalyzer 2100 system (Agilent Technologies, Santa Clara, CA, USA). The RNA samples from the same caste were equally mixed into one sample for library construction. The full-length cDNAs were synthesized using the SMARTerTM PCR cDNA Synthesis Kit (Clonetech, Palo Alto, CA, USA) using oligodT as primers and were amplified by PCR. The PCR products were purified using PB magnetic beads to remove cDNA fragments less than 1 kb in length. Subsequently, the full-length cDNAs were end-repaired and connected with SMRT adapters, then purified with PB magnetic beads again to obtain the sequencing libraries. Finally, the libraries were sequenced using PacBioSeque II (Pacific Biosciences of California, Inc., Menlo Park, CA, USA).

### 4.3. Analysis of the Raw Data

The raw sequencing data were preprocessed using the SMRTlink v10.1 (Pacific Biosciences of California, Inc., Menlo Park, CA, USA) software, and the full-length transcripts were obtained by Iso-Seq analysis process. The single molecule sequencing polymerase reads were split into subreads, where subreads with lengths less than 50 bp or greater than 15,000 bp were filtered; the remaining subreads were considered clean data. The subreads obtained from the same polymerase reads formed circular consensus sequences (CCS) after self-correction of errors. The CCS sequences were classified by detecting concatemer (chimeric sequence) and 5′ and 3′ sequencing primers, to find full-length non-concatemer (FLNC) sequences. The FLNC sequences were used for subsequent analysis.

### 4.4. Identification of Genes and Transcripts

The corrected FLNC sequences were aligned to the *A. mellifera* reference genome version. Amel_HAv3.1 (Available online: ftp://ftp.ncbi.nlm.nih.gov/genomes/all/GCA/003/254/395/GCA_003254395.2_Amel_HAv3.1/ (accessed on 21 June 2022).) using GMAP v2021-05-27 software (Department of Bioinformatics Genentech, Inc., South San Francisco, CA, USA) [52] to obtain their precise positions on the genome. According to the alignment position of each FLNC sequence, the genes (loci) and transcripts (isoforms) were identified. If two FLNC sequences mapped in the same direction and had more than 20% overlap and at least one exon had more than 20% overlap, they were considered to be transcripts from the same gene. Redundant and low-reliability transcripts from the same gene were removed, and the remaining sequences were considered the final non-redundant transcript datasets. 

The obtained gene loci were compared with the annotated loci of the *A. mellifera* reference genome version Amel_HAv3.1 through the GMAP v2021-05-27 software (Department of Bioinformatics Genentech, Inc., South San Francisco, CA, USA), and the sequenced genes were referred to as new genes if they met any of the following criteria: (1) no overlap or less than 20% overlap with the annotated genes; (2) more than 20% overlap with the annotated genes, but with the opposite orientation. The transcripts obtained by PacBio sequencing were compared with the annotated genes of *A. mellifera* reference genome, and the transcript was considered as a new one if one or more new splicing sites existed in the transcript, or if the transcript and the annotated gene were not both single exons. Functional annotation of new genes and transcripts was performed by searching against the NR, GO, KEGG, COG/KOG and Swiss-Prot databases using Diamond v2.0.7 software (University of Tübingen, Tübingen, Germany) [53].

### 4.5. Identification of Alternative Splicing Events, lncRNAs and APA Sites

Alternative splicing events, including exon skipping (ES), intron retention (IR), alternative donor site (AD), alternative acceptor site (AA) and mutually exclusive exon (MEE), were classified and counted by Astalavista v3.2 software (Johns Hopkins University School of Medicine, Baltimore, MD, USA) [54].

To identify lncRNAs, the transcript sequences of new genes and known genes were compared with NR, KOG, KO and Swiss-Prot databases to filter out possible coding sequences. The coding potential of the remaining sequences was further evaluated with the CPAT v1.2.4 (Mayo Clinic College of Medicine, Rochester, MN, USA) [55], CNCI v2.0 (Chinese Academy of Sciences, Beijing, China) [56], CPC2 vbeta (Peking University, Beijing, China ) [57] and PLEK v1.2 (Xidian University, Xi’an, Shanxi, China ) [58] softwares. All sequences with coding potential were then filtered and the remaining sequences were considered the final noncoding RNA datasets. The APA sites of each gene were detected using Tapis v1 (Colorado State University, Fort Collins, Colorado, USA) [59] software with default parameters and conserved elements near the poly (A) sites were predicted using MEME v5.3.3 software (University of Queensland, Brisbane, Queensland, Australia) [60].

### 4.6. Identification of DETs and DEGs

The RNA-seq data of *A. mellifera* queens, workers and drones from the 2-day and 4-day larvae stages were downloaded from NCBI; all transcripts obtained from this study, as well as partial transcripts from NCBI, were used as reference sequences to screen for differentially expressed transcripts. The clean reads obtained from RNA-seq were mapped to the non-redundant full-length transcripts using Bowtie2 v2.3.5 (Johns Hopkins University, Baltimore, Maryland, USA) [61] and the expression level of each transcript in each sample was calculated. DESeq2 v1.22.2 (Harvard School of Public Health, Boston, MA, USA) [62] was used for differential expression significance analysis and the |log_2_ (fold change)| > 1, FDR < 0.05 was used as the screening criterion. To identify DEGs, reads from all transcripts of the same gene were used to count the expression level of each gene and the screening criterion of DEGs was the same as DETs.

### 4.7. RT-PCR

Transcripts from five randomly selected genes were chosen for experimental validation using RT-PCR. Total RNAs were extracted from newly emerged queen, worker and drone and were reverse transcribed to cDNA. Primers were designed to amplify all the detected transcripts at the same time based on full-length sequences using *Primer* Premier 5.0 (Table S8). PCR conditions were as follows: pre-denaturation at 95 °C for 5 min; 30 amplification cycles of denaturation at 95 °C for 30 s, 58.5 °C for 45 s, 72 °C for 45 s and final elongation at 72 °C for 10 min. The PCR amplification products were detected on 1% agarose gel and followed by Sanger sequencing. 

## Figures and Tables

**Table 1:** Summary of the PacBio sequencing data.

Sample	Total Bases (Gbp)	Number of Polymerase Reads	Number of Subreads	Number of CCSs	Number of FLNCs	Average Length of FLNCs (bp)
Queen	64.80	1,100,885	43,491,193	795,183	556,221	1504
Worker	59.74	1,042,133	40,055,924	735,061	514,591	1511
Drone	65.49	1,126,347	40,663,357	792,515	546,907	1651

## Data Availability

The subreads obtained by PacBio sequencing has been submitted to the Sequence Read Archive (SRA) database and are available from NCBI under the BioProject number PRJNA941379.

## Supplementary Materials

The following supporting information can be downloaded at: https://www.mdpi.com/article/10.3390/ijms24065827/s1.
