# Supplementary material for: A Global Survey of the Full-Length Transcriptome of Apis mellifera by Single-Molecule Long-Read Sequencing
Source: Int J Mol Sci. 2023 Mar 18;24(6):5827. doi: 10.3390/ijms24065827 (PMC10059051; doi:10.3390/ijms24065827)
Supplement: Supplementary file 1 [file ijms-24-05827-s001.zip › figure & table captions.pdf]

Figure S1. Distribution of polyread lengths in the queen, worker and drone libraries.

Figure S2. Distribution of subread lengths in the queen, worker and drone libraries.

Figure S3. The number of alternative splicing events of each type in queen, worker and drone.

Figure S4. Distribution of the number of APA sites per gene.

Table S1. The list of all the 116535 transcripts.

Table S2. The function annotation of all the 116535 transcripts.

Table S3. *Summary of the* annotation results of all the transcripts and new genes.

Table S4. The unique transcripts in queen, worker and drone.

Table S5. GO enrichment of the unique transcripts in queen, worker and drone.

Table S6. The DETs and DEGs between three castes at 2 day and 4 day larvae stages.

Table S7. The samples from different developmental time points and organs/tissues of *A. mellifera*.

Table S8. Primers for verification of alternative splicing events.
