# Supplementary material for: A Global Survey of the Full-Length Transcriptome of Apis mellifera by Single-Molecule Long-Read Sequencing
Source: Int J Mol Sci. 2023 Mar 18;24(6):5827. doi: 10.3390/ijms24065827 (PMC10059051; doi:10.3390/ijms24065827)
Supplement: Supplementary file 1 [file ijms-24-05827-s001.zip › TableS3.pdf]

Table S3 Summary of the annotation results of all the transcripts and new genes.

Database	Transctipt	New gene
Total	116,535 (100.00%)	18,915 (100.00%)
NR	92,438 (79.32%)	2958 (15.64%)
GO	59,272 (50.86%)	1567 (8.28%)
KO	67,958 (58.32%)	1512 (7.99%)
KOG	29,912 (25.67%)	216 (1.14%)
Swiss-Prot	70,442 (60.45%)	1098 (5.80%)
Unannotated	24,058 (20.64%)	15,948 (84.31%)
