# Supplementary material for: A Global Survey of the Full-Length Transcriptome of Apis mellifera by Single-Molecule Long-Read Sequencing
Source: Int J Mol Sci. 2023 Mar 18;24(6):5827. doi: 10.3390/ijms24065827 (PMC10059051; doi:10.3390/ijms24065827)
Supplement: Supplementary file 1 [file ijms-24-05827-s001.zip › TableS7.pdf]

Table S7 The samples from different developmental time points and organs/tissues of *A. mellifera*.

Sample	Developmental time points and organs/tissues
Queen	0h egg, 24h egg, 48h egg, 72h egg, 2d larvae, 4d larvae, 5d larvae, 3d pupae, 5d pupae, 7d pupae, new emerged queen, sexually mature queen; and antenna, brain, hypopharyngeal gland, muscle, leg, middle intestine, skin, ovary of new emerged queen.
Worker	0h egg, 24h egg, 48h egg, 72h egg, 2d larvae, 4d larvae, 6d larvae, 3d pupae, 6d pupae, 8d pupae, new emerged worker, nurse, forger; and antenna, brain, hypopharyngeal gland, muscle, leg, middle intestine, skin of new emerged queen
Drone	0h egg, 24h egg, 48h egg, 72h egg, 2d larvae, 5d larvae, 3d pupae, 8d pupae, 12d pupae, new emerged drone, sexually mature drone; and antenna, brain, hypopharyngeal gland, muscle, leg, middle intestine, skin, testis of new emerged drone
