# Supplementary material for: A Global Survey of the Full-Length Transcriptome of Apis mellifera by Single-Molecule Long-Read Sequencing
Source: Int J Mol Sci. 2023 Mar 18;24(6):5827. doi: 10.3390/ijms24065827 (PMC10059051; doi:10.3390/ijms24065827)
Supplement: Supplementary file 1 [file ijms-24-05827-s001.zip › TableS8.pdf]

Table S8 Primers for verification of alternative splicing events

Gene	Primer sequences
NC_037638.1.1016	F: CACTTCGGCAACGCTGGAT<br>R: ACTTTCTTGGATTGAAATCCTTGGC
NC_037640.1.1269	F: AATACATCAAGTTATGAGG<br>R: GCATTTCACTTATTGGTTGTCC
NC_037640.1.1279	F: AGCATTGGGAGCATCACATCC<br>R: TGCGGCTCCTTCCATACGAT
NC_037641.1.755	F: CGATACGCCGAGGACGAAAG<br>R: CTCGTTCGGTCGTTGCAGTT
NC_037641.1.72	F: TGGTCCTTCGGTACAACGTG<br>R: CATGTGTATCCGCATCATTCGT
