# Supplementary figures and images for: A Global Survey of the Full-Length Transcriptome of Apis mellifera by Single-Molecule Long-Read Sequencing
Source: Int J Mol Sci. 2023 Mar 18;24(6):5827. doi: 10.3390/ijms24065827 (PMC10059051; doi:10.3390/ijms24065827)

Queen

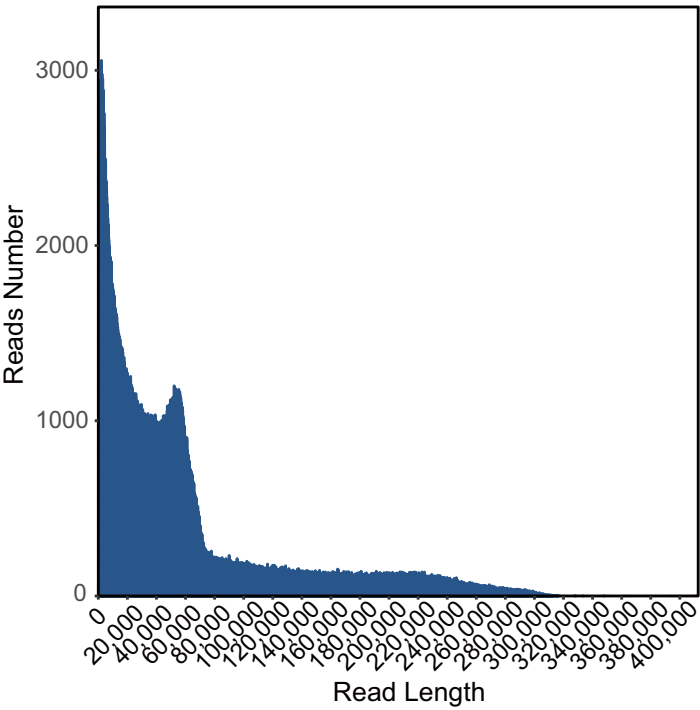

Worker

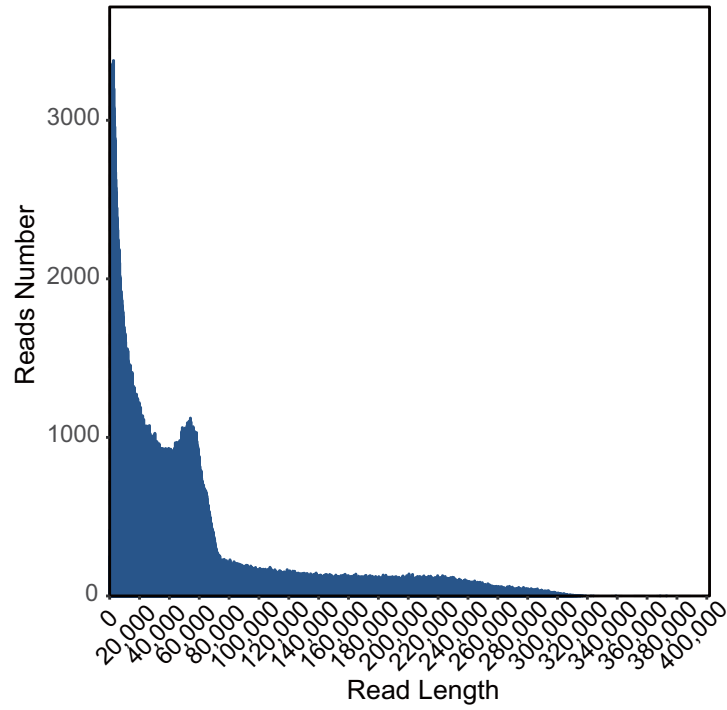

Drone

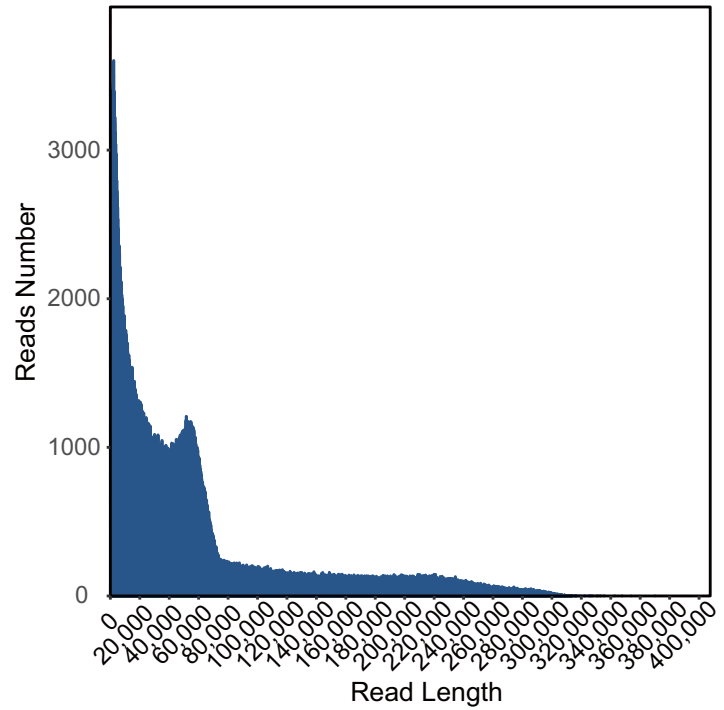

Supplement: Supplementary file 1 [file ijms-24-05827-s001.zip › FigureS1.pdf]

Queen

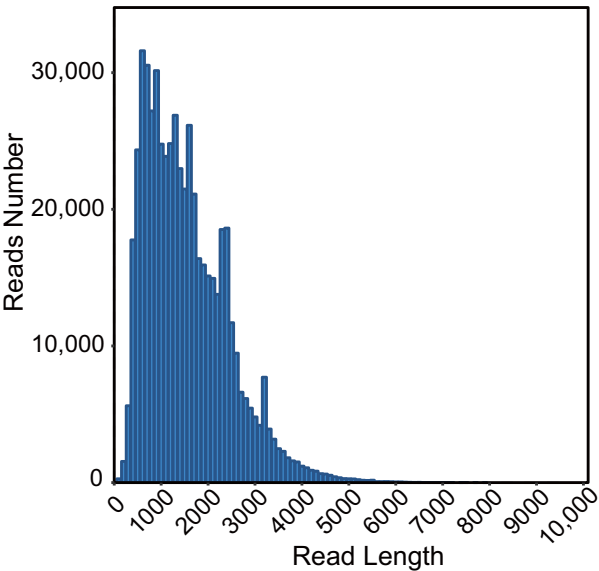

Worker

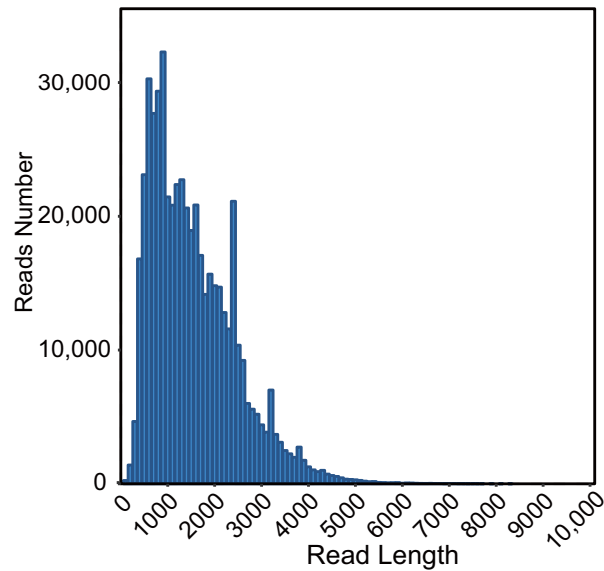

Drone

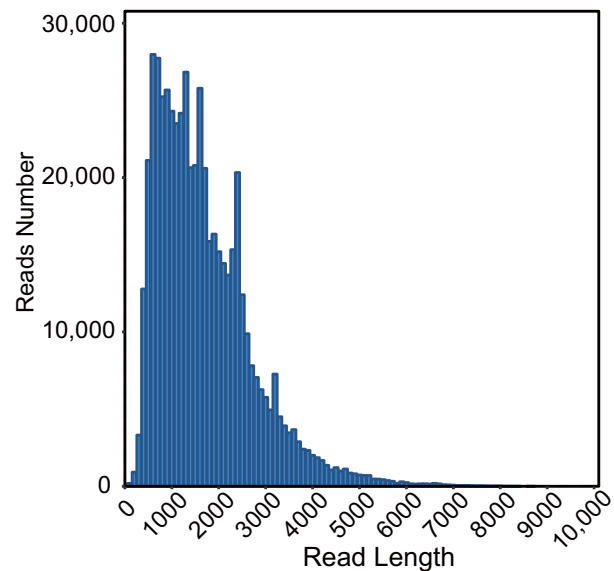

Supplement: Supplementary file 1 [file ijms-24-05827-s001.zip › FigureS2.pdf]

Queen

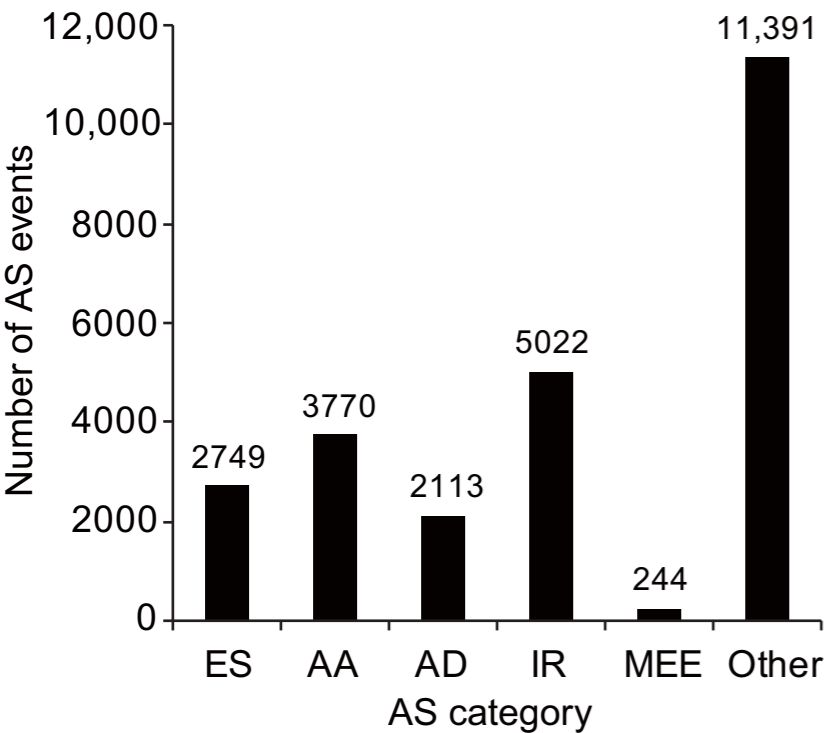

Worker

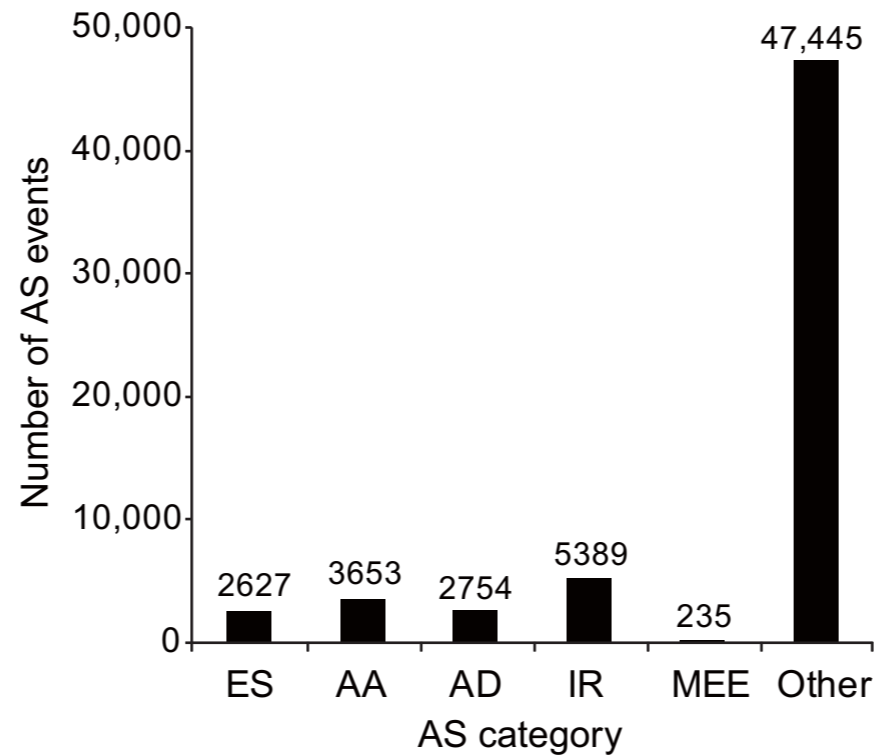

Drone

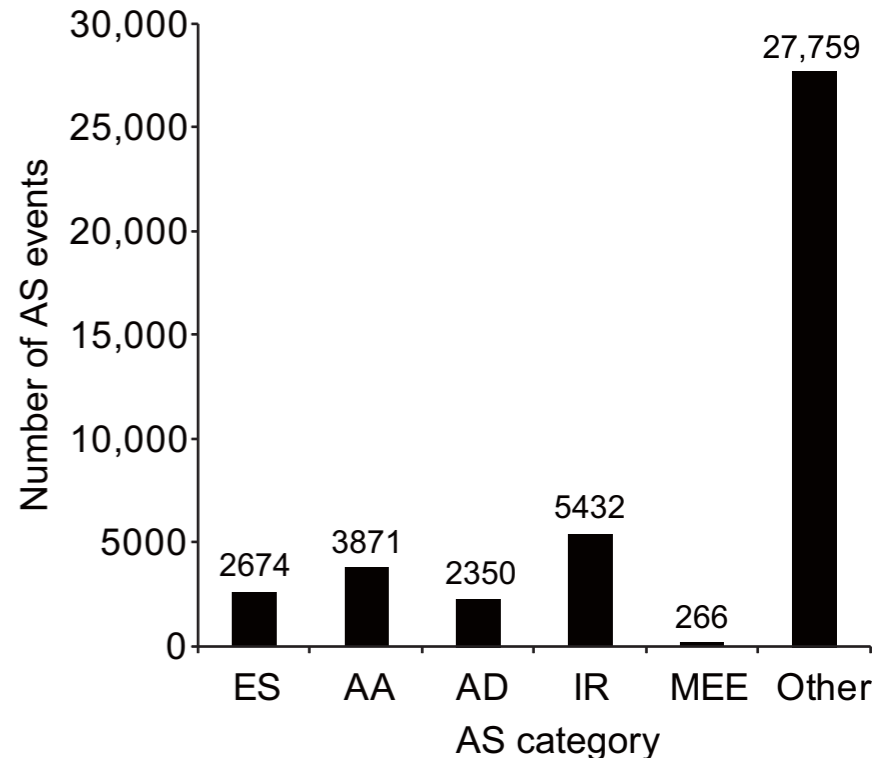

Supplement: Supplementary file 1 [file ijms-24-05827-s001.zip › FigureS3.pdf]

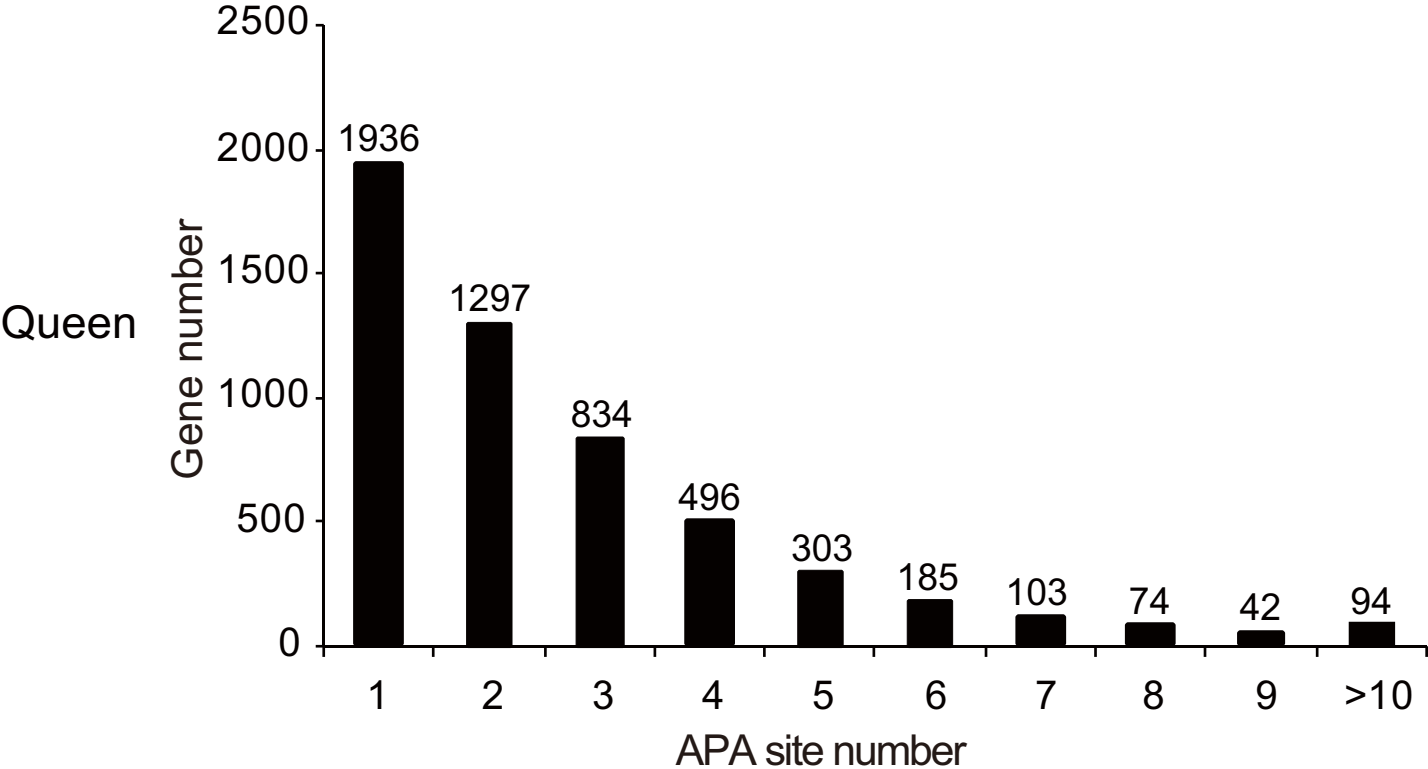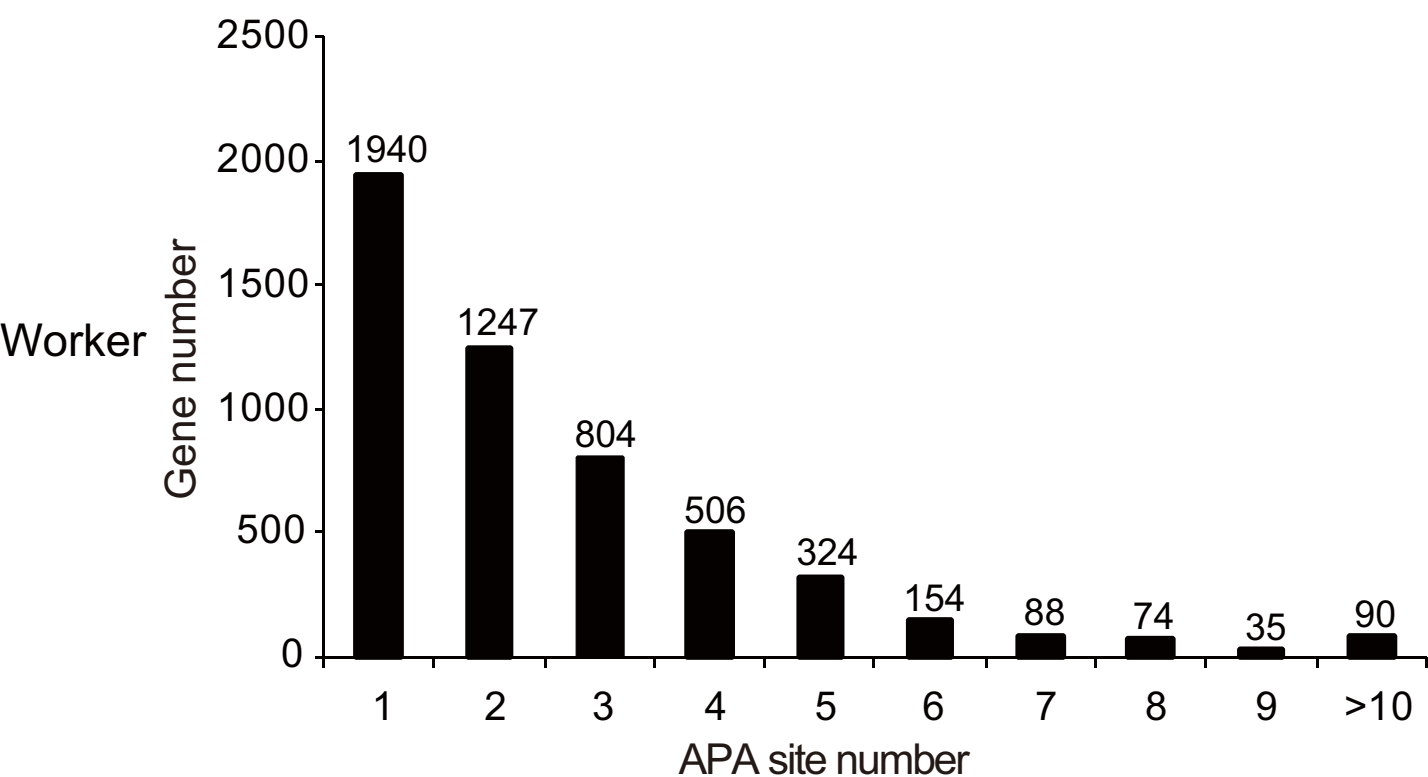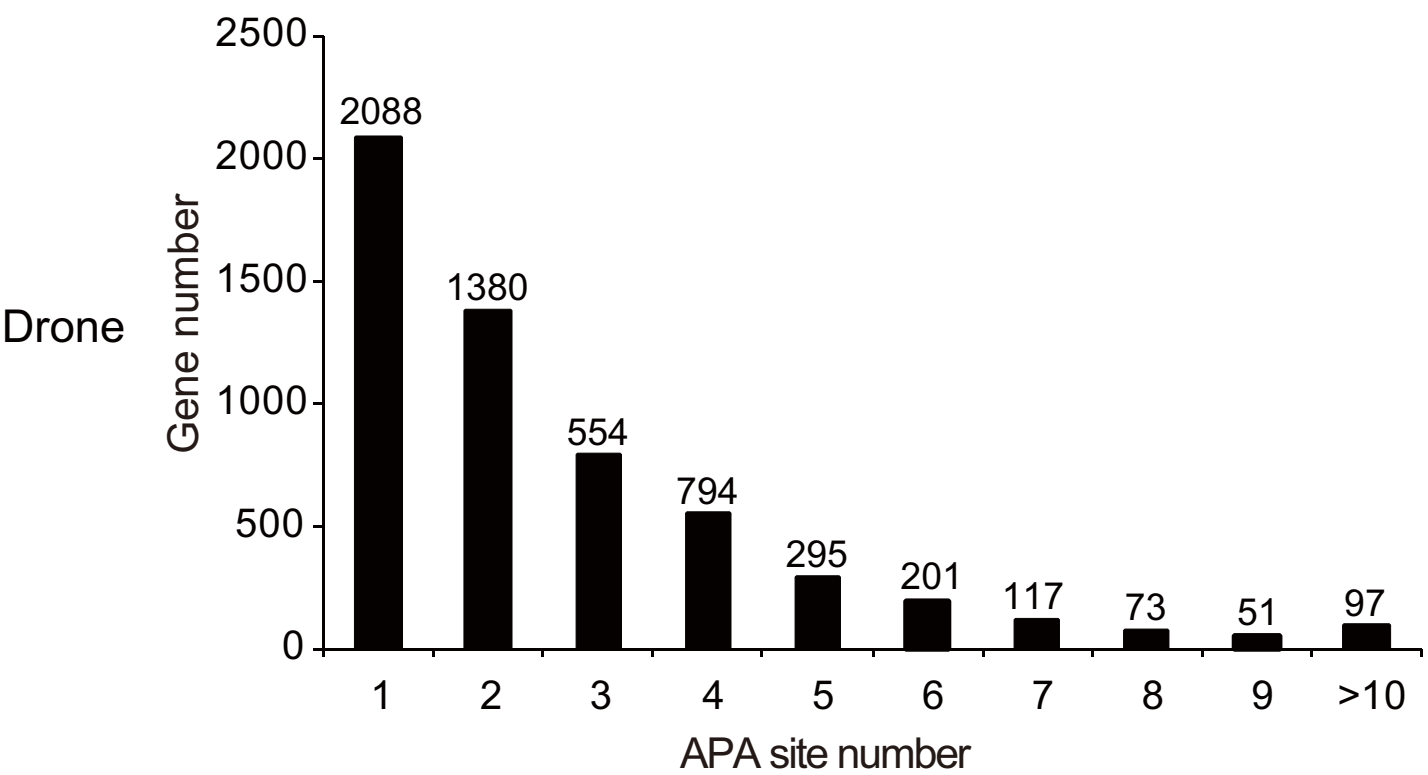

Supplement: Supplementary file 1 [file ijms-24-05827-s001.zip › FigureS4.pdf]
